# Effectiveness and cost-effectiveness of a telehealth intervention to support the management of long-term conditions: study protocol for two linked randomized controlled trials

**DOI:** 10.1186/1745-6215-15-36

**Published:** 2014-01-24

**Authors:** Clare L Thomas, Mei-See Man, Alicia O’Cathain, Sandra Hollinghurst, Shirley Large, Louisa Edwards, Jon Nicholl, Alan A Montgomery, Chris Salisbury

**Affiliations:** 1 Centre for Academic Primary Care, NIHR School for Primary Care Research, School of Social and Community Medicine, University of Bristol, Canynge Hall, 39 Whatley Road, Bristol BS8 2PS, UK; 2 School of Health and Related Research (ScHARR), University of Sheffield, Regent Court, 30 Regent Street, Sheffield S1 4DA, UK; 3 NHS Direct, Strawberry Fields, Berrywood Business Village, Tollbar Way, Hedge End, Southampton SO30 2UN, UK; 4 Nottingham Clinical Trials Unit, University of Nottingham, C Floor, South Block, Queen’s Medical Centre, Nottingham NG7 2UH, UK

**Keywords:** Cardiovascular disease risk, Depression, Randomized controlled trial, Telehealth

## Abstract

**Background:**

As the population ages, more people are suffering from long-term health conditions (LTCs). Health services around the world are exploring new ways of supporting people with LTCs and there is great interest in the use of telehealth: technologies such as the Internet, telephone and home self-monitoring.

**Methods/Design:**

This study aims to evaluate the effectiveness and cost-effectiveness of a telehealth intervention delivered by NHS Direct to support patients with LTCs. Two randomized controlled trials will be conducted in parallel, recruiting patients with two exemplar LTCs: depression or raised cardiovascular disease (CVD) risk. A total of 1,200 patients will be recruited from approximately 42 general practices near Bristol, Sheffield and Southampton, UK. Participants will be randomly allocated to either usual care (control group) or usual care plus the NHS Direct Healthlines Service (intervention group). The intervention is based on a conceptual model incorporating promotion of self-management, optimisation of treatment, coordination of care and engagement of patients and general practitioners. Participants will be provided with tailored help, combining telephone advice from health information advisors with support to use a range of online resources. Participants will access the service for 12 months. Outcomes will be collected at baseline, four, eight and 12 months for the depression trial and baseline, six and 12 months for the CVD risk trial. The primary outcome will be the proportion of patients responding to treatment, defined in the depression trial as a PHQ-9 score <10 and an absolute reduction in PHQ-9 ≥5 after 4 months, and in the CVD risk trial as maintenance or reduction of 10-year CVD risk after 12 months. The study will also assess whether the intervention is cost-effective from the perspective of the NHS and personal social services. An embedded qualitative interview study will explore healthcare professionals’ and patients’ views of the intervention.

**Discussion:**

This study evaluates a complex telehealth intervention which combines evidence-based components and is delivered by an established healthcare organisation. The study will also analyse health economic information. In doing so, the study hopes to address some of the limitations of previous research by demonstrating the effectiveness and cost-effectiveness of a real world telehealth intervention.

**Trial registration:**

Current Controlled Trials: Depression trial ISRCTN14172341 and cardiovascular disease risk trial ISRCTN27508731.

## Background

As the population ages, the priority for the UK National Health Service (NHS) is increasingly to help people manage long-term conditions (LTCs). Over 15 million people in England have a LTC, and the treatment of LTCs accounts for 70% of total healthcare expenditures. Therefore, improvements in LTC management could have major benefits in terms of patient health, quality of life and use of NHS resources [1]. It is acknowledged that there is a need to redesign services both to cope with the increasing number of people needing healthcare and to improve the standard of care being offered. The Department of Health has developed a strategy for improving the care of patients with LTCs that is based on promoting better health by supporting self-care and providing responsive, high-quality services and case management for those with the greatest needs [2].

There is strong international interest in using new technologies such as the Internet, text messaging, telephone support or remote monitoring to help patients with LTCs [3]. There is a large body of research in this area, including the Whole System Demonstrator project, a recent large-scale cluster randomized controlled trial (RCT) conducted in the United Kingdom [4]. The research evidence has been summarised in a number of systematic reviews of telehealth delivery for a variety of LTCs [5-18]. These reviews show that the evidence of its effectiveness is stronger for some conditions (for example, heart failure) than for others (for example, diabetes). Furthermore, there is good evidence that telehealth is feasible and can improve health-related behaviour but a lack of evidence about mechanisms of action, health outcomes, value for money, patient satisfaction, impact on the use of healthcare services and acceptability. The Healthlines Study aims to address these limitations.

The two RCTs—an economic evaluation and an embedded qualitative study—described in this protocol are being conducted in the context of a wider programme of research known as the Healthlines Study. This trial is a 5-year project funded by the National Institute for Health Research (NIHR) Programme Grants for Applied Research scheme (grant reference RP-PG-0108-10011). The programme commenced in November 2009 and is a collaboration between NHS Direct; the Universities of Bristol, Sheffield, Manchester and Southampton; and the Royal College of Surgeons in Ireland. The overall aim of the programme is to design, develop and evaluate telehealth interventions for patients with LTCs to be delivered by NHS Direct.

## Methods/Design

### Outline of trial design

**Figure 1:**
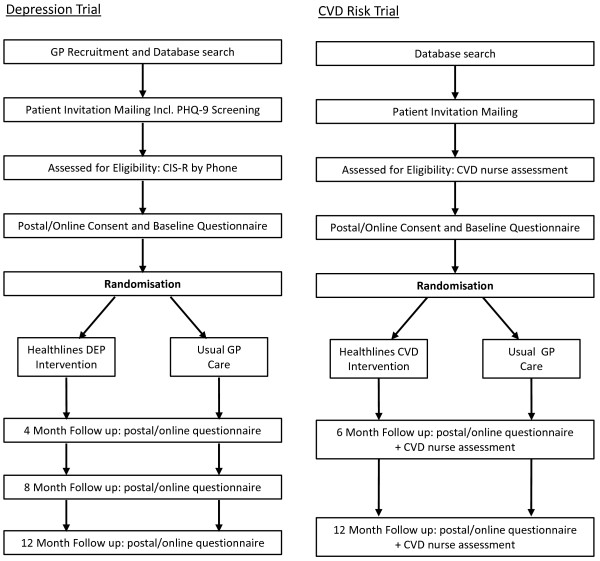
**Flowchart of participant recruitment and follow-up procedures.** CIS-R, Clinical Interview Schedule–Revised; CVD, Cardiovascular disease; GP, General practice; PHQ-9, Patient Health Questionnaire.

We describe two linked, multicentre, parallel two-arm, individually randomized trials involving two different patient groups: those with depression and those with raised cardiovascular disease (CVD) risk. The study infrastructure, including participating general practices, research staff and intervention staff, and the underlying theoretical basis for the intervention are common across both trials. However, the specific content of the intervention package, data analysis and reporting are distinct. Hereinafter these trials are referred to as the “depression trial” and the “CVD risk trial”. Figure 1 shows a flowchart summarising participant recruitment and follow-up procedures for each of the trials. The first two general practices recruited into the study will act as a run-in phase during which study procedures will be tested and refined before patients are recruited from the remaining practices. Alongside the RCTs, we will also carry out an economic evaluation and an embedded qualitative study to explore the acceptability of the intervention.

### Trial aim and objectives

The aim of the trials is to determine the clinical efficacy and cost-effectiveness of an NHS Direct–delivered telehealth intervention to support patients with two exemplar LTCs: depression and raised cardiovascular risk. Specifically, the study will address the following research questions:

1. Does the intervention, in addition to usual primary care, improve condition-specific clinical outcomes compared with usual care alone?

2. Does the intervention have any effect on other patient outcomes, including quality of life and satisfaction with care?

3. What is the cost-effectiveness of the intervention in each condition?

4. What is the acceptability of and compliance with the intervention, and what are the facilitators of and barriers to its delivery?

### Settings

The trial settings will comprise approximately 42 general practices in the environs of Bristol, Sheffield and Southampton. Two practices will participate in the run-in phase, and the remainder will be involved in the main trials. The final number of practices will depend on achievement of participant recruitment targets.

### Inclusion criteria

In both trials, participants will be required to have access to a telephone (landline or mobile), the Internet and an e-mail address for personal use. Additional inclusion criteria for the CVD risk trial are (1) age between 40 and 74 years on the date of invitation to participate, which is the age range for which CVD risk scores have been validated and the same age range used for the NHS Health Checks vascular screening programme; (2) 10-year risk of a cardiovascular event ≥20% calculated using the QRISK2 score [19]; (3) at least one of the following modifiable risk factors: (a) current systolic blood pressure (BP) ≥140 and suitability for home BP monitoring, (b) body mass index ≥30 and (c) currently smoking. Additional inclusion for the depression trial are (1) age ≥18 years on the date of invitation to participate, (2) confirmed diagnosis of depression using the Clinical Interview Schedule–Revised (CIS-R) [20] and (3) Patient Health Questionnaire (PHQ-9) score ≥10 [21]. Patients who meet the inclusion criteria for both the raised CVD risk and depression trials will be invited to take part in the depression trial only. All inclusion criteria must be met for inclusion in the study.

### Exclusion criteria

Exclusion criteria applying to both trials are listed below:

•Bipolar disorder

•Psychotic illness

•Dementia or substantial cognitive impairment

•Severe learning disability

•Substance dependency

•Receiving palliative care

•Significant suicide risk

•General practitioner’s determination that participation would cause distress (for example, due to recent bereavement)

•Inability to communicate verbally in English sufficiently to receive telephone-based support delivered in English. (Patients who can communicate verbally in English but are unable to read English will be eligible, provided that they have a family member or friend who is willing and able to translate written materials (such as information sheets, consent forms and online material) for them.

Additional exclusion criteria for the CVD risk trial are (1) established diagnosis of CVD, defined as history of heart attack, angina, heart failure, stroke or transient ischaemic attack; (2) currently pregnant or planning to become pregnant within the next 12 months; (3) patients who will be invited to participate in the NHS Health Checks Programme during the period of the trial (in cases where the local Primary Care Trust has requested this); and (4) patients with atrial fibrillation for whom high BP is their only modifiable risk factor.

Additional exclusion criteria for the depression trial are (1) currently receiving case management from a specialist mental health worker; (2) currently receiving face-to-face, telephone or computerised cognitive-behavioural therapy (CBT) or similar psychotherapy; and (3) having given birth in the previous 12 months. An individual will be excluded if any of the criteria are met.

### Recruitment procedures

#### Initial identification and sampling

In the CVD risk trial, potentially eligible individuals will be identified using a search of general practice computerised patient records. Patients whose records suggest they have a raised CVD risk and at least one modifiable risk factor will be selected. *Raised CVD risk* is defined as QRISK2 10-year risk score ≥20% calculated using risk factor information extracted from the patient’s record.

In the depression trial, potentially eligible patients will be identified using two methods: records search and direct healthcare professional (HCP) referral. The search will include patients who have consulted the doctor for depression, low mood or other similar symptoms or who have been prescribed antidepressants within the previous 2 months. It will exclude individuals with any of the study exclusion criteria.

With direct healthcare professional (HCP) referrals, the general practitioner (GP) or other HCP will identify potentially eligible participants during patients’ consultations. The GP or other HCP will explain the study to the patient and provide an invitation letter and the participant information sheet. If the patient is interested in taking part, the GP or other HCP will complete a referral checklist and, with the patient’s permission, pass the patient’s contact details to the local research team.

In both trials, a random sample of the potentially eligible patients derived from the records search or all of the identified patients, whichever is the smaller group, will be selected. The initial plan is to invite 250 and 300 patients per practice for the CVD and depression trials, respectively, but this number will be revised based on actual recruitment rates in the run-in phase and early main trial practices to ensure that recruitment targets are met. In both trials, GPs will be asked to review the list of potentially eligible patients and exclude any patients felt to be unsuitable (for example, due to recent bereavement).

#### Invitations and reminders

Practice staff will send patients identified in a records search an invitation letter and participant information sheet. Patients interested in participating will be asked to respond directly to the research team, providing contact details and consent for eligibility screening. Those who do not wish to take part will be asked to return a decline form. Practice staff will send one postal reminder to individuals who have not yet responded 3 weeks after the initial mailing and also have the option of using telephone reminders if they wish. In the depression trial, interested patients will also be asked to complete and return a PHQ-9 questionnaire at this stage, which will be used to make a preliminary assessment of the patient’s eligibility for the study.

#### Confirmation of eligibility

Patients who reply to express an interest in participating will be contacted by a member of the local research team by telephone. The researcher will clarify with the patient what taking part in the study will involve and answer any questions. The patient’s ability to access the Internet and e-mail will also be confirmed. Further eligibility screening will then proceed in the two trials as described next.

In the CVD risk trial, patients will be asked to attend an appointment at their GP practice with a pretrained practice nurse or healthcare assistant (HCA). The nurse or HCA will take BP readings using an OMRON M3 upper-arm BP monitor (product code 031201; OMRON Healthcare UK Ltd, Milton Keynes, UK), as well as measure height and weight, and collect other information necessary to calculate CVD risk using the QRISK2 algorithm. Smoking status will be assessed by patient self-report and validated by measurement using a carbon monoxide monitor (COmpact Smokerlyzer (product code 01420000); Bedfont Scientific, Maidstone, UK). A nonfasting blood sample will be taken to determine the patient’s total cholesterol to high-density lipoprotein cholesterol ratio. If the patient has had this test conducted within the previous 3 months, however, the existing result will be used and the blood test will not be repeated. The CVD risk assessment information will then be sent to the research team, and used to calculate a QRISK2 10-year risk score and verify the presence of at least one modifiable risk factor. Patients will be contacted by e-mail or telephone to inform them of their eligibility status.

In the depression trial, the research team will telephone patients who have a total PHQ-9 score ≥10 on the questionnaire to conduct further eligibility screening. During the call, the researcher will conduct the CIS-R screening assessment to establish whether the patient has a confirmed diagnosis of depression. This measure has been validated for telephone completion [22]. The researcher will then immediately confirm eligibility.

#### Consent, baseline assessment and randomisation

Patients confirmed as eligible will be asked to complete a consent form and a baseline assessment questionnaire. This can be done online or by post. Once both have been completed and received, patients will be randomly allocated in a 1:1 ratio to receive either (1) usual care plus the NHS Direct Healthlines Service (intervention group) or (2) usual care alone (control group). Patients will be allocated using an automated web randomisation system to ensure concealment from research staff. Randomisation will be stratified by location of recruitment and minimised by practice and baseline CVD risk or depression score, retaining a probabilistic element by using a computer-generated random number sequence. The minimisation categories for QRISK2 score are 20.0 to 24.9, 25.0 to 29.9 and ≥30.0, and for PHQ-9 score they are 10 to 14, 15 to 19 and ≥20 [21].

#### Communication following allocation

Participants in both trials will be notified of their allocation by e-mail. Their GP will also be informed of their participation in the study, their allocation and their baseline scores. Participants in the intervention group will be sent a link to the NHS Direct Healthlines Service website, where they will be provided with additional information about the study intervention. The participants’ details will be passed to the NHS Direct intervention team, and a member of the intervention team will contact the patient by telephone to conduct an initial assessment. Participants in the intervention group in the CVD risk trial who have raised BP and do not also have atrial fibrillation will be offered a home BP monitor to use during the study. Pretrained practice staff will provide a short training session to ensure the participant knows how to use the monitor.

### Trial run-in phase

The run-in phase of the trial will commence recruitment 1 month ahead of the main trials. Two general practices will be involved, and each will aim to recruit twenty patients to the depression trial and twenty to the CVD risk trial. The purposes of the run-in phase are to test study recruitment and follow-up procedures and to allow adjustments before the main trials if required. These steps will also allow intervention staff to develop their skills in using the intervention software and treatment protocols. In order to provide intervention staff with a larger number of participants to work with during the run-in phase, allocation in this phase will be at a ratio of 3:1 in favour of the intervention group. Data collection and intervention schedules will be the same for run-in phase participants as in the main trials, so data provided by these participants will be included in the final trial analysis unless substantial changes to the trial protocol are required between the run-in phase and the main trial.

### MRC START in Healthlines substudy

The Medical Research Council Systematic Techniques for Assisting Recruitment to Trials (MRC START) study is a programme of research funded by the Medical Research Council Methodology Programme, which is designed to develop the conceptual, methodological and logistical framework for nested studies and to assess their feasibility [23]. The Healthlines Study is acting as a host trial for one of the MRC START recruitment interventions. Its aim is to test the impact of an enhanced patient information sheet and invitation letter on recruitment rates [24]. We aim to carry out this substudy in up to four GP practices at the Bristol site only.

### NHS Direct Healthlines intervention

#### Intervention staff

The Healthlines intervention will be delivered by a team of NHS Direct health information advisors (HIAs). These staff will be existing NHS Direct employees based within an existing NHS Direct call centre. All the HIAs will have a minimum of a diploma-level qualification or equivalent experience in a healthcare or social care setting. They will also have experience working for NHS Direct in providing expert health and medication information. The HIAs will receive 3 weeks of training prior to delivering the Healthlines intervention. This training will include classroom sessions, hands-on training and side-by-side call reviews with expert training staff. It will cover health coaching skills, training on how to introduce and support the online or workbook CBT package for depression, how to use CVD risk intervention software and condition-specific knowledge and medicines training, with the latter being delivered by expert pharmacists. Throughout delivery, the Healthlines advisors will have access to clinical advice and management support as required. Staff performance will also be monitored using a rigorous call review process.

#### Hours and mode of operation

The NHS Direct Healthlines Service will be available from Monday to Friday from 10 AM to 8 PM and on Saturday from 10 AM to 2 PM. The service will provide an initial assessment followed by regular telephone contacts at prearranged appointment times. Where possible, all contacts with an individual patient will be made by the same staff member in order to promote continuity of care and build rapport. The work of intervention staff will be supported by computer software, which will be used to guide the content of the telephone calls and create a record of what was discussed. In addition, patients will be able to request a call-back between their scheduled appointments via the intervention website or by leaving a telephone voice mail message.

#### Initial assessment

An initial telephone assessment will be conducted to provide further information about the NHS Direct Healthlines Service and the treatment options available to the participant. Relevant medical history and medication use will also be recorded. The next scheduled appointment will be arranged, and a report will be sent to the participant’s GP.

#### Intervention content

**Figure 2:**
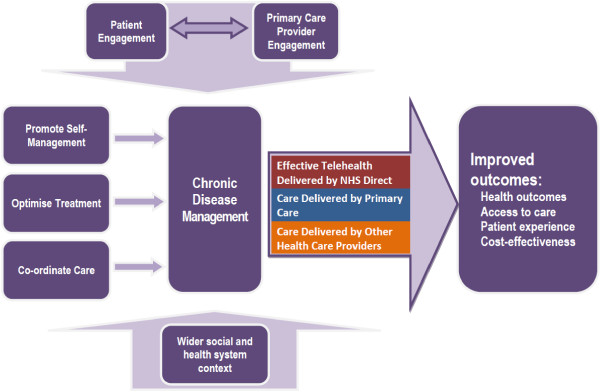
Healthlines intervention conceptual model.

The content of the intervention was developed to reflect the conceptual model (Figure 2). The model was created by the Healthlines research team based on findings from a systematic review of the characteristics of effective telehealth interventions, a qualitative study of patient and staff experiences of related initiatives [25] and a patient survey of attitudes toward different types of telehealth interventions. These studies were conducted in the earlier phase of the Healthlines research programme. Appropriate and evidence-based priorities for intervention were also identified by reviewing UK treatment guidelines for depression and CVD risk factors and relevant research literature, including Cochrane reviews. Furthermore, we compared our findings about important components of the intervention model with other related models, such as the Chronic Care Model [26]. The final conceptual model highlights the need to promote self-management (including using established approaches such as goal-setting, self-monitoring, information-sharing, decision-making, relapse prevention and regular review), optimisation of treatment (particularly titration of medications following protocols), coordination of care between providers and methods designed to enhance the engagement of patients and GPs.

The intervention is designed to address each of the components of the model, with the particular types of help offered to each participant being tailored to their needs and preferences. The core of the intervention consists of regular telephone calls from a HIA, who supports the participant in setting and addressing their goals by using advice derived from computerised protocols and support scripts. These scripts frequently guide the participant to relevant resources that are available on the Internet, including information from reliable sources (particularly NHS Choices [27]), interactive programmes such as computerised CBT and relevant apps and widgets (for example, to help with giving up smoking). Having identified a relevant resource, the HIA will e-mail the participant a link to the relevant website or resource or will send them information by post. The HIA will also review the participant’s progress with regard to each goal during subsequent calls, seeking to enhance the participant’s motivation.

The intervention also includes access to an individualised password-protected page of the NHS Direct Healthlines Service web portal. This portal provides more information about the service and about the participant’s health condition (CVD risk or depression), enables participants with raised BP to record their BP readings and has links to other resources.

Development of the NHS Direct Healthlines Service incorporated findings of a usability and acceptability study conducted by NHS Direct. The overall aim of that study was to explore the utility, usability and acceptability of the proposed services for people with depression and raised CVD risk. Three deliberative panels comprising eleven patients were convened, and ten in-depth interviews were conducted with patients with either depression or raised CVD risk. The patients were recruited from one GP practice in the Southampton area. The study explored patient perspectives about the usefulness, benefits and drawbacks of the intervention and how the service could be used by patients to achieve specified goals within the context of its specified purpose. Accessibility issues related to the service were also discussed to help the research team understand potential barriers to using the service.

In the CVD risk trial, the software and telephone scripts were adapted from effective interventions developed by Bosworth and colleagues at Duke University (Durham, NC, USA) [28,29]. The content and language of this original intervention was modified where appropriate to make it consistent with UK treatment guidelines and the NHS environment and to incorporate additional components written by the NHS Direct Clinical Content Team and CS. The software guides the delivery of a series of modules during 12 telephone contacts approximately 4 weeks apart over a 12-month period. The modules cover a wide variety to topics associated with the management of raised CVD risk. The software is designed so that the intervention staff covers topics which are relevant to the patient during each telephone contact, and the content of later encounters reflects the topics that were covered in earlier calls. A list of the modules is given below:

•Knowledge about cardiovascular risk and healthy lifestyles

•Medication and side effects review

•Blood pressure medication optimisation

•Home blood pressure monitoring

•Statin medication review

•Support for medication adherence

•Smoking and nicotine replacement therapy

•Diet

•Weight loss and orlistat use

•Alcohol use

A key aspect of the CVD risk intervention is support for home BP monitoring. Patients with a systolic BP ≥140 who do not have atrial fibrillation will be offered an OMRON M3 home BP monitor. This device is a basic monitor validated for home use by the European Society of Hypertension International Protocol [30]. Participants will be given instructions on how and when to record their BP and how to enter their readings on the Healthlines web portal. Participants are requested to take BP readings twice daily for the first week and weekly thereafter. The web portal calculates average readings initially over the previous 6 days (if the participant has entered at least four readings) and then over the previous 6 weeks. The participant is then automatically advised whether their BP is above their target, when to take their BP again and what to do if their BP is too high or too low. Target BP is based on UK National Institute for Health and Care and Excellence (NICE) guidelines [31], but it can be modified for individual patients by their GPs. The HIA will review the patient’s BP readings during each monthly call. If the readings are above target, the HIA will advise the patient to see his or her GP and send the GP a letter asking the GP to review the patient’s treatment, including a copy of the relevant NICE guidelines about recommended steps for intensifying treatment. A copy of this letter will also be available to the patient via the web portal.

In the depression trial, participants will be offered access to the Living Life to the Full Interactive programme, supported by regular telephone contact. Living Life to the Full Interactive is an online interactive multimedia programme that delivers CBT-based treatment for depression (or comorbid depression and anxiety). It was developed by Chris Williams of the University of Glasgow, UK [32,33]. It involves six self-directed sessions completed approximately every 2 weeks. The programme is offered to Healthlines participants either as an online interactive programme provided by Media Innovations Ltd (http://www.llttfi.com/) or by giving them the *Overcoming Depression and Low Mood: A Five Areas Approach* workbook to use [34]. As in the CVD risk trial, at the core of the intervention is regular telephone calls from HIAs at the NHS Direct Healthlines Service designed to support participants in making use of the materials available mainly via the Internet. The telephone scripts used during each contact were written by the NHS Direct Clinical Content Team and CS and incorporate protocols for providing support to participants in using and applying Living Life to the Full Interactive. In addition they include modules covering monitoring of depression symptoms, optimising and stepping up treatment in cases of inadequate response, medication adherence, exercise and alcohol use. Participants in the depression trial receive seven telephone calls at approximately fortnightly intervals over the course of 4 months, then two further contacts made once every 3 months over the course of 6 months. Participants will also have access to the Healthlines web portal, which includes a link to the Living Life to the Full Interactive programme. If they wish, they can also access from the web-portal Big White Wall [35], a digital mental health system with a 24/7 professionally managed peer support network.

In addition to the regular telephone calls and support for using resources available online, the intervention incorporates several other features designed to address components of the underlying theoretical model. Because one recognised problem with previous telehealth interventions was low patient engagement, intervention-mapping was used to identify features that would act as barriers or facilitators to patient engagement. As a result, the intervention has been designed to address perceived barriers and highlight factors that would facilitate and reinforce engagement. One factor discovered during the developmental work to enhance patient engagement was the importance of support from a named person rather than from an impersonal call centre. Therefore, participants will be offered follow-up from the same HIA as much as possible. Previous telehealth interventions have also had problems related to engagement of primary care clinicians [25]. The present research aims to address this problem by framing the intervention as supportive and working with primary care clinicians rather than duplicating their efforts or competing with them. Accordingly, letters are regularly sent to the GPs about each patient’s progress during the course of the intervention. Letters making recommendations about treatment are also accessible to patients, which reinforces patient engagement and may make it more likely that recommendations will be acted upon.

#### Intervention completion

Patients can remain in receipt of the intervention for up to 12 months. The final patient contact will include a review of progress, information about other ongoing sources of support and creation of a final summary to be sent to the GP. Upon completion of interventions, participants will be returned to the sole care of their GPs. This transition will be clearly communicated to both the participants and their GPs.

### Outcome measurement

#### Primary outcomes

In both trials, the primary outcome will be the proportion of patients who respond to treatment. In the CVD risk trial, response to treatment is defined as the maintenance or reduction of 10-year cardiovascular risk estimated on the basis of QRISK2 score [20] after 12 months. Because CVD risk increases with age, maintaining 10-year risk over 12 months requires an improvement in at least one modifiable risk factor. Throughout the study, QRISK2 score will be calculated using the same version of the QRISK2 algorithm provided as a batch processor to the research team by the authors of QRISK2. QRISK2 outcome scores will be calculated by changing age and the modifiable risk factors only. Variables such as diagnosis of diabetes or atrial fibrillation and whether the patient has been prescribed BP-lowering medications will be held constant using baseline values. This is because it is possible that the extra support provided by the intervention may result in an increased number of patients in the intervention arm receiving a diagnosis or starting medication, which in turn would inflate risk scores in this group only. In the depression trial, response is defined as a PHQ-9 score <10 and an absolute reduction in PHQ-9 ≥5 after 4 months [21,36].

#### Secondary outcomes and process measures

Secondary outcomes and process measures are listed in Table 1. These measures will be included in both trials unless otherwise indicated.

**Table 1:** **Secondary outcomes and process measures **^**a**^

**Outcomes**	**Measures used**
Depression score (depression trial only) as a binary outcome at 8 and 12 months and as a continuous variable at 4,8 and 12 months	PHQ-9 [21]
Ten-year CVD risk score (CVD risk trial only) as a binary outcome at 6 months and as a continuous variable at 6 and 12 months	QRISK2 [19] variable obtained by clinical assessment
Quality of life	EQ-5D-5 L [37]
Patient satisfaction	Items constructed for present research and taken from Y4Q1 GP Patient Survey
Patient perceived access to care	Items adapted from TMQ [38] and items constructed for present research
Physical activity	Health-directed behaviour domain of heiQ [39]
Individual cardiovascular risk factors that is blood pressure, cholesterol, smoking status, weight, BMI (CVD risk trial only)	Clinical assessment
Diet (CVD risk trial only)	STC [40] anglicised for present use
Use of telehealth	Items constructed for present research
Internet use and experience	Items constructed for present research
Self-management skills and self-efficacy	Self-monitoring and insight, constructive attitudes and approaches, skill and technique acquisition, and healthcare services navigation domains of heiQ [39]
Medication adherence (depression trial asked only about antidepressant medication; CVD trial asked about antihypertensive and cholesterol-lowering medications)	Morisky Medication Adherence Scale [41]
Health literacy	Items adapted from eHEALS [42]
Care coordination	Subscales adapted from a measure of continuity questionnaire [43]
Anxiety (depression trial only)	GAD-7 [44]
Resource use	Items constructed for present research
Demographics	Various items taken from the Y4Q1 GP Patient Survey, 2001 census and Health Survey for England, 2007

^a^*BMI*, Body mass index; *CVD*, Cardiovascular disease; *eHEALS*, eHealth Literacy Scale; *GAD-7*, Generalized Anxiety Disorder 7-item scale; *heiQ*, Health Education Impact Questionnaire; *PHQ-9*, Patient Health Questionnaire; *STC*, Starting the Conversation; *TMQ*, Treatment Motivation Questionnaire.

### Data collection and follow-up

Data will be collected from self-report questionnaires, primary care medical records, NHS Direct Healthlines Service records and clinical measurement. Information will be collected at baseline from all participants, but the timing of follow-up data collection will differ between the two trials (as detailed below). The primary method of patient self-report data collection will be via online questionnaires. However, alternative completion methods, including on paper or by telephone, will be offered to maximise response rates. The quality of life measure (EQ-5D-5 L) will not be available for online completion, owing to licensing and technical restrictions, and thus will be completed on paper by all participants. Reminders communicated by e-mail, telephone and post will be used if participants do not return their questionnaires. The baseline questionnaire will include sociodemographic characteristics, comorbidities, current treatments, employment status and all secondary outcome measures. Follow-up questionnaires will include primary and secondary outcome measures and use of healthcare resources. Details regarding medication prescriptions and primary care contacts will be collected from primary care medical records for all participants 12 months after randomisation. For those in the intervention groups in both trials, information about the type, number and length of contacts with intervention staff will be collected from NHS Direct Healthlines Service records. In addition, anonymised information, including age, gender and ethnicity (both trials) and CVD risk score and component data (CVD risk trial only), will be obtained from practice records of invited patients who decided not to participate in the study.

In the CVD risk trial, self-report questionnaires will be completed at three time points: at baseline and 6 and 12 months after randomisation. At each of these time points, patients will also be asked to attend an appointment with a nurse at their GP practice. The nurse will collect CVD risk factor information, including a blood test to measure cholesterol (at baseline and 12 months only), BP, weight and smoking status (all time points). This information will be used to calculate the individual QRISK2 score.

In the depression trial, self-report questionnaires will be completed at four time points: at baseline and 4, 8 and 12 months after randomisation. The PHQ-9 questionnaire will be completed at each of these time points. At baseline, it will be completed as part of an initial screening questionnaire by post; at all other time points, it will be included with the follow-up questionnaire and therefore completed online or by post. Participants who do not return their follow-up questionnaires will be contacted by post and telephone to ask them to complete the PHQ-9 alone.

### Statistical considerations

#### Sample size

This study is based on analysis of data from 240 patients in each of the intervention and control groups for both the CVD risk and depression trials. The primary outcomes for both CVD and depression are binary, indicating response to the intervention. Assuming equal numbers in each trial arm for analysis and that all other parameters in a sample size estimate remain constant, the detectable between-group difference for a binary outcome is maximised when the overall proportion in the trial defined as responding is 50% (for example, 57% responding to NHS Direct Healthlines interventions and 43% responding to usual care). As the overall proportion responding increases or decreases from 50%, so the detectable difference decreases. Thus, with a sample size for analysis of 240 per arm, the absolute detectable difference between the trial arms with a 5% two-sided α and 80% power is 14 percentage points or less (1.7 equivalent odds ratio). With a 1% α and 90% power, it is 18 percentage points or less (2.1 equivalent odds ratio). For this trial, there is no accepted minimum clinically important difference. Therefore, the sample size was chosen pragmatically, taking into account the size of effect that would be likely to influence policy and practice and which might be feasible using this intervention.

Regarding the CVD risk trial, a previous study reported 38% of treated hypertensive patients 60 to 80 years of age in a control group had a reduced absolute cardiovascular risk at the 12-month follow-up examination [45]. Regarding the depression trial, response to treatment using the PHQ-9 is defined as a score <10 and a reduction of at least 5 points. In a previous trial involving a similar patient group, the response in the control group was approximately 30% [46]. If the proportion in the control arm in either of our trials is in the 30% to 38% range, 240 participants per arm for analysis will have 80% power (5% α) and 90% power (1% α) to detect differences of 13 and 18 percentage points, respectively.

Assuming 20% noncollection of primary outcome data at follow-up, it will be necessary to recruit 300 patients in each of the intervention and control groups for each trial, or 1,200 patients in total.

#### Descriptive analysis

The analysis and presentation of each trial will be carried out in accordance with Consolidated Standards of Reporting Trials guidelines. Appropriate descriptive statistics will be used to compare characteristics of invited patients who did or did not agree to take part and eligible patients who were randomized or not randomized. We will also examine the balance of patient characteristics at baseline across the trial groups.

#### Primary analysis

By using intention-to-treat analyses, we will compare groups using logistic regression models adjusted for baseline values of the outcome and stratification/minimisation variables, paying attention to 95% confidence intervals as well as *P*-values. Sensitivity analyses will be conducted using standard techniques to impute missing data.

#### Secondary and subgroup analyses

Analysis of secondary outcomes, the effect of adherence to the intervention and preplanned subgroup analyses for the primary outcome will be conducted using appropriate multivariable regression models. The subgroups of interest are age, sex and baseline CVD risk or PHQ-9 score. Subgroups broken down by the type of modifiable risk factor at baseline (for example, high BP, obesity, smoker) will be analysed in the CVD risk trial only. Additional subgroups of interest may also be identified by the process evaluation. Analysis of additional subgroups will be agreed upon with the Data Monitoring Committee and the Trial Steering Committee, and all analyses performed will be reported. A full statistical analysis plan will be developed and agreed upon with the Trial Management Group, the Data Monitoring Commitee and the Trial Steering Committee prior to any analyses.

#### Protection against bias

Allocation will be concealed by use of a remote automated system. Blinding of participants, HCPs and researchers is not possible. We recognise that patients in the control group may be able to access similar information from other websites, including NHS Choices, although we think that such usage will be much less than in the intervention group because the latter will be directed to these resources by NHS Direct Healthlines Services staff. We will collect patient self-report information about the use of other relevant web-based resources by all participants.

### Research governance

Management, operational and academic aspects of the trials are the responsibility of the Trial Management Group, which meets approximately every 6 weeks. Conduct of the trials will also be overseen by an Independent Trial Steering Committee and an Independent Data Monitoring Committee. The whole research programme is also overseen by the Healthlines Programme Management Group, which includes all the coapplicants on the programme grant and patient and public representatives. The University of Bristol is the study sponsor, and the study has been approved by the National Research Ethics Service Committee South West–Frenchay (Reference 12/SW/0009).

### Economic evaluation

The aim of the economic evaluation will be to estimate the costs and benefits of the NHS Direct Healthlines intervention in managing patients with raised CVD risk or with depression and to assess the cost-effectiveness of the telehealth packages in these contexts. There will be two stages of this evaluation: (1) patient-level evaluations covering the period of the trial and (2) modelling of future costs and benefits to cover the lifetime of the trial population. The patient-level evaluations will be carried out prospectively alongside the depression and CVD risk trials. These evaluations will take into account the perspectives of the healthcare provider (NHS) and personal social services (PSS), patients and lost productivity due to time taken off from work.

#### Identifying resource use

NHS resource use will include any care related to the participant’s condition. For patients with depression, primary and community care is wide-ranging, thus we will include all contacts with GPs, other doctors (such as community psychiatrists), all nurses based in GP practices and in the community, and other HCPs (such as counsellors, psychologists and occupational therapists). Relevant medications will be identified using prespecified British National Formulary (BNF) codes [47]. Relevant secondary care and PSS use will be identified by patients as being sought out “because of mental health or emotional problems”. For patients with raised CVD risk, resource use will be restricted to contacts related to high BP, high cholesterol, giving up smoking or overweight. NHS resource use will include all primary care, medication and secondary care.

Personal out-of-pocket expenditures will vary by condition. For patients with depression, this will include the use of private healthcare and therapies, exercise specifically aimed at improving mental health and domestic help and travel. For those with raised CVD risk, this will include the use of private healthcare and therapies, home BP monitors, smoking advice and strategies, exercise aimed at reducing BP and cholesterol, weight loss programmes and travel. Resources used in setting up and delivering the intervention will be identified during the trial and documented by NHS Direct.

#### Measuring resource use

Where possible, data on primary care use and medication will be extracted from GPs’ notes. Data on other resource use, including personal expenditures and time taken off from work, will be collected using the questionnaires administered as part of the data collection programme within the trial follow-up. These questionnaires will be completed at 4, 8 and 12 month by patients with depression and at 6 and 12 months by those with raised CVD risk. Data on the resources required to set up and run the Healthlines intervention will be recorded as part of the trial process.

#### Valuing resource use

NHS resources will be valued using nationally recognised sources such as Curtis [48], the Department of Health Tariff [49] and the BNF [47]. Patients will self-report personal costs. Time taken off from work will be valued using the Annual Survey of Hours and Earnings published by the Office for National Statistics [50]. The intervention will be costed using data collected during the trial and supplemented by information supplied by NHS Direct. The startup costs will be identified and estimated separately from the running costs, and the cost of each element of the intervention will be estimated separately.

#### Sensitivity analysis

We will address any areas of uncertainty in structural assumptions using a series of one-way sensitivity analyses.

#### Cost-effectiveness

Costs will be compared with benefits measured using the primary outcomes of QRISK2 for patients with raised CVD risk and PHQ-9 for those with depression. In both cases, we will compare costs with quality-adjusted life years (QALYs) derived from the EQ-5D-5 L. QALYs will be calculated by valuing the health states on the EQ-5D-5 L using the “cross-walk” between the EQ-5D-3 L value sets and the EQ-5D-5 L as recommended by the EuroQol group [37]. These sets are based on the valuations provided by a sample of the UK population. Incremental cost-effectiveness ratios will be obtained for each patient group. Uncertainty regarding these ratios will be captured by using the bootstrapping technique and estimating the net monetary benefit.

In the second stage of the economic evaluation, we will use data from the trial along with other data in the literature to develop simulation models similar to that used in the Department of Health modelling of vascular checks [51]. These models will be used to estimate cost per QALY over the life of the trial population and indicate parameters of importance in implementation of the programme at the national level.

Parameters for both models will include the uptake, effectiveness and cost of the different components of the intervention package. They will also include the prevalence of the conditions, probability of disease progression (for example, developing severe depression or experiencing a cardiovascular event), mortality associated with each health state, age, sex, socioeconomic status and other risk factors for CVD, such as smoking. Quality of life for each health state, as well as the cost of relevant treatments, will be estimated using data from the trial and the literature. Costs and benefits will be discounted in line with current recommendations prevailing at the time [52].

Threshold analysis will be performed to identify required uptake, effectiveness and cost of the package for the intervention to be cost-effective for given levels of prevalence. We will also aim to identify subgroups of high-risk patients for whom the intervention is most appropriately targeted [53].

### Embedded qualitative study

Qualitative research undertaken alongside a trial can consider the acceptability of the intervention as well as facilitators and barriers to the delivery of, use of and compliance with the intervention [54]. The perspectives of both patients and HCPs can be sought.

#### Design, methods and setting

We will undertake a qualitative interview study with HCPs involved in delivering care to patients with the two conditions and with patients in the intervention groups of the trials. Face-to-face semistructured interviews with patients and a mix of face-to-face and telephone interviews with HCPs involved in delivering care will be conducted.

#### Sampling

To include practices serving populations with different levels of deprivation, six of the general practices involved in the trials will be selected to take part in the embedded qualitative study: one in Bristol, one in Southampton and four in Sheffield. Between 15 and 25 interviews will be undertaken with HCPs offering care to participants within the trial, including NHS Direct staff, GPs and practice nurses from the six selected practices, and 20 to 30 participants in the intervention arm of the trial will also be interviewed. A purposive sampling strategy will be used by condition to ensure half of interviewees have depression and half have risk factors for CVD. Next, maximum variation sampling will be used so that patients of different socioeconomic backgrounds, gender and age are interviewed.

#### Recruitment

HCPs in selected practices and NHS Direct Healthlines Service staff will be sent a letter of invitation, information sheet and consent form, which they will return if they wish to participate. Sampled patients participating in the trials who, as part of the trial consent, have agreed that they could be approached to consider taking part in an interview will be sent an information sheet and consent form.

#### Data collection

HCP interviews will be conducted at different times during the trial period. Interviews will commence after the trial has been running for 6 months. Patients will be interviewed at least 6 months after randomisation. The focus of the interviews will be on the intervention: its perceived utility, problems that arise, issues that enhance or hinder its operation in practice, and adherence. An interview topic guide will be used. Interviews with HCPs will take place at their workplaces, or by telephone if that is more convenient. Interviews with patients will take place at their homes or at their general practice, depending on their preference.

#### Analysis

Interviews will be recorded and transcribed verbatim. Framework analysis will be used. The thematic framework will be informed partly by issues that arise in early phases of the programme and partly by themes that emerge from the interviews.

We also plan to combine elements of the qualitative study with some of the quantitative results within a mixed-methods process evaluation to explore issues related to implementation, fidelity to the intervention model, factors which appeared to act as barriers to and facilitators of the success of the intervention and which aspects of the intervention the patients and practitioners felt were most and least successful.

## Discussion

There is a need for new, efficient, effective interventions to support the growing number of people living with LTCs. This study will evaluate one such intervention delivered using the telephone, Internet and home monitoring. Previous research on similar telehealth interventions have been criticised for the lack of theoretical underpinning and for failing to adequately evaluate the impact on health outcomes, patient satisfaction, use of health services and cost of delivery. We aim to address these criticisms. Our intervention is theory-based and combines a number of intervention components for which there is already some evidence of effectiveness (for example, computerised CBT or home BP monitoring) [29,55-57]. Use of a conceptual model to guide intervention design is a strength of the study, as it will allow us to explore the mechanism of action. In addition, carrying out two linked trials provides the opportunity to evaluate the intervention in two contrasting patient groups. If the intervention is found to be effective in both patient groups, confidence in the efficacy of the underlying theoretical model would be increased, suggesting that successful interventions for other LTCs could be designed based on the same model. The study will also incorporate a variety of methods, including a qualitative interview study, economic evaluation and economic modelling, in order to maximise understanding of the findings and lessons for future commissioning and implementation. Working with NHS Direct, an existing NHS organisation with an established infrastructure and expertise in the design and delivery of telephone- and Internet-based healthcare, is a further strength of the study. Setting up this complex study within the time frame and budget of a research programme has presented a number of challenges. These challenges have included introducing a new service during a time of major NHS reorganisation and appropriately adapting software developed in the United States and integrating it with additional new software designed by NHS Direct. NHS Direct and the research team have worked hard to overcome these challenges. We feel that this large, multicentre, pragmatic study with a multimethod design will make a positive contribution to the evidence base for telehealth interventions.

### Trial status

Recruitment of general practices commenced in April 2012. Participant randomisation commenced in July 2012 and was completed on 31 July 2013.

## Abbreviations

BNF: British National Formulary; BP: Blood pressure; CBT: Cognitive-behavioural therapy; CIS-R: Clinical Interview Schedule–Revised; CVD: Cardiovascular disease; GP: General practitioner; HCA: Healthcare assistant; HCP: Healthcare professional; HIA: Health information advisor; LTC: Long-term condition; NHS: National Health Service; NIHR: National Institute for Health Research; PHQ-9: Patient Health Questionnaire; QALY: Quality-adjusted life year; RCT: Randomised controlled trial.

## Competing interests

The authors declare they have no competing interests.

## Authors’ contributions

AO’C, SH, SL, JN, CS and AM are coapplicants on the Healthlines Study programme grant. CS is the Chief Investigator with overall responsibility for the design of the programme and is the Principal Investigator for the RCT. AM was the Principal Investigator for the trial until his move to Nottingham in April 2013, and he continues as Trial Statistician with responsibility for statistical aspects of the design, conduct and analysis of the trial. AO’C is lead for the qualitative study, and SH is lead for the economic evaluation. SL is Head of Research at NHS Direct and has overseen the development of the study intervention within NHS Direct. CT and MSM (maternity cover) are the programme managers with responsibility for drafting and updating the study protocol. LE is the lead researcher at the Bristol study site with responsibility for participant recruitment and data collection. All authors are members of the Trial Management Group and therefore have been involved in the development of the trial protocol. CT created the first draft of the manuscript with assistance from MSM, CS, AM and LE. All authors reviewed and commented on the draft and approved the final version of the manuscript.
